# Characteristics of HIV-1 gp120 molecules that bind ancestor, intermediate and mature forms of VRC01-like antibodies

**DOI:** 10.1186/1742-4690-9-S2-P68

**Published:** 2012-09-13

**Authors:** M Joyce, B Zhang, T Zhou, S Moquin, X Wu, M Louder, Z Zhang, I Georgiev, J Zhu, L Shapiro, JR Mascola, GJ Nabel, PD Kwong

**Affiliations:** 1 Vaccine Research Center, NIAID/NIH, Bethesda, MD, USA

## Background

A group of highly effective neutralizing antibodies, which target the site of CD4 binding on HIV-1 gp120, have recently been identified. These antibodies – called VRC01-like antibodies – all originate from the same VH1-2*02 germline gene and, while the mature antibodies have undergone extensive maturation via non-homologous pathways, their recognition of the CD4-binding site of gp120 is similar. An efficacious vaccine that elicits VRC01-like antibodies will likely be required to bind to VH1-2*02-derived B cells to initiate their expansion and subsequent maturation, however, binding studies with reverted-ancestor VRC01-like antibodies and HIV-1 gp120 molecules typically show binding that is too weak to initiate B cell maturation.

## Methods

To identify HIV-1 gp120 molecules capable of interacting with reverted-ancestor molecules with sufficient affinity to initiate B cell maturation, we screened large panels of HIV-1 pseudoviruses for sensitivity to reverted-ancestor forms of VRC01-like antibodies. Identified HIV-1 strains (and related gp120s) were then analyzed for recognition to a panel of diverse VRC01-like antibodies.

## Results

No HIV-1 strains were identified which could be neutralized by reverted heavy chain- and light chain-ancestors of VRC01-like antibodies. Chimeric forms of the VRC01-like antibodies with reverted and mature heavy/light chain mixtures did, however, neutralize a small subset of HIV-1 isolates. Characterization of gp120s from the sensitive subset found measurable affinity to the ancestral forms of VRC01-like antibodies. In comparison, typical gp120 molecules, e.g. YU2 gp120, fail to bind low-divergent forms of the VRC01-like antibodies, i.e. those with less than 10% divergence from germline.

## Conclusion

Select strains of HIV-1 can interact with ancestral forms of VRC01-like antibodies. Defining the specific characteristics of these select strains should enable identification of gp120-derived immunogens capable of productive interactions with VH1-2*02-derived B cells.

